# Crosstalk between autophagy inhibitors and endosome-related secretory pathways: a challenge for autophagy-based treatment of solid cancers

**DOI:** 10.1186/s12943-021-01423-6

**Published:** 2021-10-27

**Authors:** Martina Raudenska, Jan Balvan, Michal Masarik

**Affiliations:** 1 Department of Physiology, Faculty of Medicine, Masaryk University, Kamenice 5, CZ-625 00 Brno, Czech Republic; 2 Department of Chemistry and Biochemistry, Mendel University in Brno, Zemedelska 1, CZ-613 00 Brno, Czech Republic; 3 Department of Pathological Physiology, Faculty of Medicine, Masaryk University, Kamenice 5, CZ-625 00 Brno, Czech Republic; 4 BIOCEV, First Faculty of Medicine, Charles University, Prumyslova 595, CZ-252 50 Vestec, Czech Republic; 5 Center for Advanced Functional Nanorobots, Department of Inorganic Chemistry, Faculty of Chemical Technology, University of Chemistry and Technology in Prague, Technická 5, CZ-166 28 Prague, Czech Republic

**Keywords:** Autophagy, Autophagy inhibitors, Cancer, Endosomes, Multivesicular bodies, Extracellular vesicles, Exosomes, Amphisomes, Non-conventional secretory pathways

## Abstract

Autophagy is best known for its role in organelle and protein turnover, cell quality control, and metabolism. The autophagic machinery has, however, also adapted to enable protein trafficking and unconventional secretory pathways so that organelles (such as autophagosomes and multivesicular bodies) delivering cargo to lysosomes for degradation can change their mission from fusion with lysosomes to fusion with the plasma membrane, followed by secretion of the cargo from the cell. Some factors with key signalling functions do not enter the conventional secretory pathway but can be secreted in an autophagy-mediated manner.

Positive clinical results of some autophagy inhibitors are encouraging. Nevertheless, it is becoming clear that autophagy inhibition, even within the same cancer type, can affect cancer progression differently. Even next-generation inhibitors of autophagy can have significant non-specific effects, such as impacts on endosome-related secretory pathways and secretion of extracellular vesicles (EVs). Many studies suggest that cancer cells release higher amounts of EVs compared to non-malignant cells, which makes the effect of autophagy inhibitors on EVs secretion highly important and attractive for anticancer therapy. In this review article, we discuss how different inhibitors of autophagy may influence the secretion of EVs and summarize the non-specific effects of autophagy inhibitors with a focus on endosome-related secretory pathways. Modulation of autophagy significantly impacts not only the quantity of EVs but also their content, which can have a deep impact on the resulting pro-tumourigenic or anticancer effect of autophagy inhibitors used in the antineoplastic treatment of solid cancers.

## Introduction

Autophagy is a highly evolutionarily conserved mechanism best known for its role in organelle and protein turnover, cell quality control, and metabolism. Lysosome-mediated degradative autophagy provides a source of nutrients and energy by digestion of cytoplasmic elements and serves for the clearance of toxic protein aggregates and defective organelles [1]. This recycling pathway can also profoundly affect cellular specialization and differentiation [2], protein trafficking, and unconventional secretion [3, 4]. Three types of autophagy have been observed in mammalian cells: macroautophagy, microautophagy, and chaperone-mediated autophagy. During microautophagy the lysosomal membrane insulates the autophagic cargo directly, whereas, during macroautophagy, double-membrane structures called autophagosomes are formed to deliver autophagic cargo to endosomes or lysosomes. Macroautophagy also participates in the specific degradation of organelles during mitophagy, ribophagy, or pexophagy. Chaperone-mediated autophagy involves the selective degradation of KFERQ-like motif-bearing proteins supplied to the lysosomes via chaperone HSC70 and other cochaperones (e.g. CHIP, HOP, and heat shock protein 40). Internalization of cargo into lysosomes is managed via the receptor lysosome-associated membrane protein type 2A (LAMP2A) [5]. In this review article, we will focus on macroautophagy (hereafter referred to as autophagy).

**Fig. 1:**
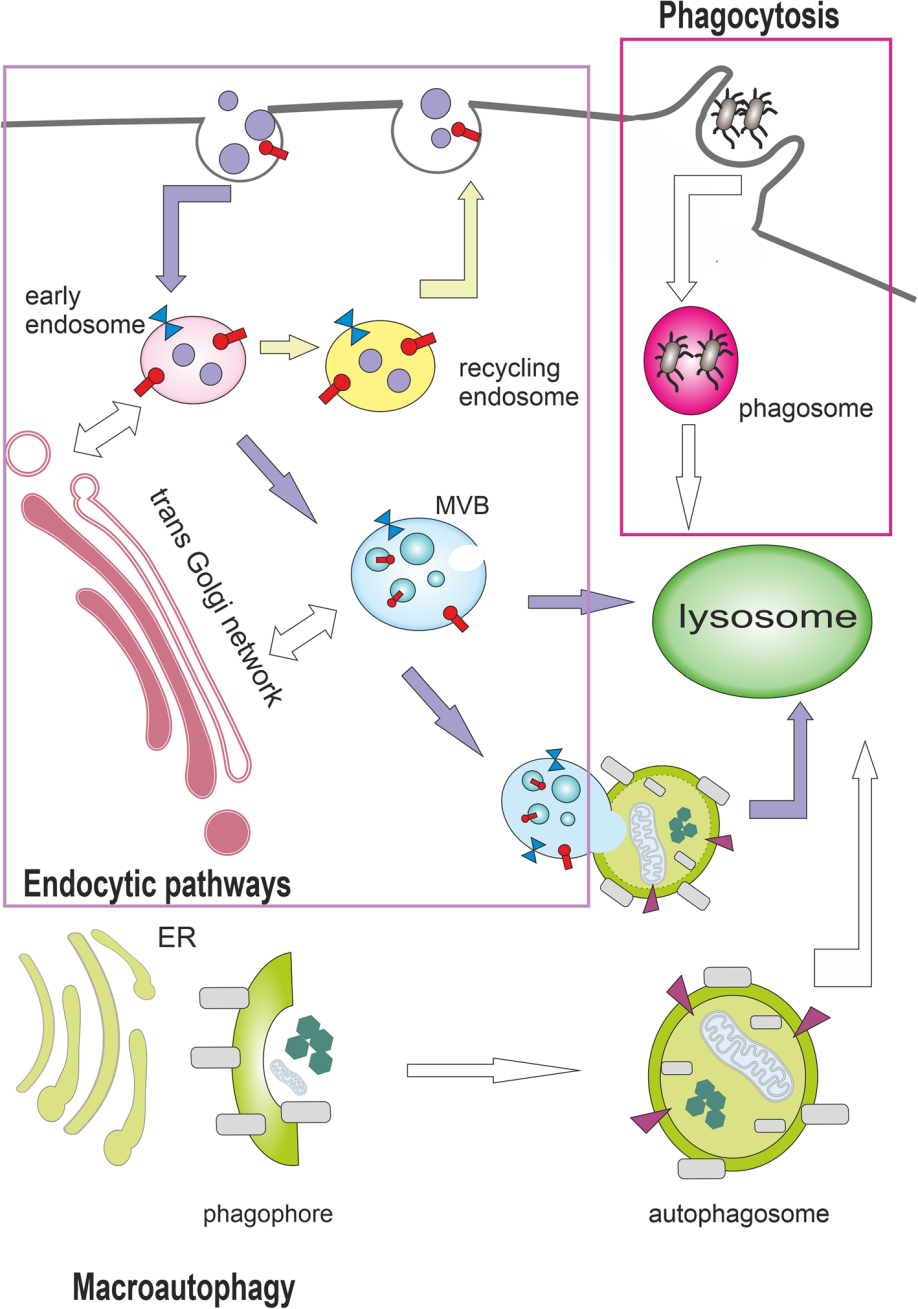
Autophagy and endocytic pathways can culminate in lysosomes. Endocytosis enables the transport of substances from the external environment and includes the clathrin-dependent pathway as well as clathrin-independent pathways such as phagocytosis. Phagocytosis is the endocytosis of large molecules or intact microorganisms. Protrusions of plasma membrane surround and internalize the extracellular cargo into single-membrane structures called phagosomes, which are then transported to the lysosome for degradation. Endocytosis (clathrin-mediated endocytosis is shown here) involves invagination of the plasma membrane and biogenesis of small intracellular vesicles that contain constituents of the plasma membrane and extracellular components. These small vesicles fuse and establish the compartment called the early endosome (EE). EE cargo can be recycled to the plasma membrane via recycling endosomes, transported to or from the Golgi apparatus via the retromer complex, or routed to lysosomes via multivesicular bodies (MVBs). During macroautophagy, double-membrane structures called autophagosomes are formed to deliver autophagic cargo to lysosomes or to fuse with MVBs. Autophagy and endocytic pathways cooperate at some stages and share many components of the molecular machinery

Endocytosis is the process by which cells can sequester substances from the external environment by engulfing them in vesicles. Endocytosis includes the clathrin-dependent pathway as well as clathrin-independent pathways such as phagocytosis, pinocytosis, raft-mediated endocytosis, and ARF6-dependent internalization. As well as autophagy, endocytosis can culminate into lysosomal degradation, but here the cargo is internalized from the plasma membrane, not from the cytoplasm [6]. After internalization, the cargo is sorted by highly dynamic compartments, called early endosomes (EEs), marked by unique adaptor proteins, effector proteins, and small Rab GTPases such as RAB4, RAB5, early endosomal antigen-1 (EEA1), VPS34, and SNAREs. EEs are the major cellular sorting platform as they can mature into endosomes destined for various cellular fates. EE cargo can be recycled to the plasma membrane via recycling endosomes, transported to or from the Golgi apparatus via the retromer complex, or routed to lysosomes via multivesicular bodies (MVBs)/late endosomes [7]; see Fig. 1. Autophagy and endocytic pathways cooperate at some stages and share many components of the molecular machinery.

Recent studies also show that there are many interconnections between autophagy, exosome/amphisome biogenesis, and exocytosis of extracellular vesicles (EVs) [3, 4]. To release exosomes and/or amphisomes, several steps need to be performed such as the biogenesis of intraluminal vesicles (ILVs) in MVBs, transport of MVBs to autophagosomes or the plasma membrane and fusion of MVBs and/or amphisomes with the plasma membrane [8]. These steps are deeply affected by molecules of autophagy machinery [9, 10] including many Rab GTPases such as RAB7, RAB11, RAB35, RAB27A, RAB27B and the vesicle-associated membrane protein 7 (VAMP7) [11]. RAB7 and RAB11 also participate in autophagosome formation and RAB7 has a key role in autophagosome maturation (for further details see the chapter Autophagy and MVBs) [12]. Consequently, autophagy can have both stimulatory or inhibitory effects on the secretion of extracellular vesicles (EVs) and these effects will probably be deeply context-dependent. This can partially explain the double-edged sword character of autophagy in cancer progression. In this review article, we discuss how different inhibitors of autophagy may influence the secretion of EVs and summarize the non-specific effects of autophagy inhibitors with a focus on endosome-related secretory pathways. Modulation of autophagy significantly impacts not only the quantity of EVs but also their content which can have a deep impact on the resulting pro-tumourigenic or anticancer effect of autophagy inhibitors used in antineoplastic treatment of solid cancers.

## Basic molecular mechanism of degradative macroautophagy

Macroautophagy (hereafter referred to as autophagy) is a process in which double-membrane vesicles (autophagosomes) are formed around a segment of the cytoplasm. Once autophagosomes are formed, they can either fuse with lysosomes and form autolysosomes, or they can bring together organelles of endosomal origin to form amphisomes with a single limiting membrane [13–15].

Autophagosome formation goes through five main stages — initiation, nucleation, elongation, fusion, and cargo degradation. The detailed molecular mechanism of autophagy has been extensively reviewed in Klionsky et al. [16], therefore, we present here only the basic molecular mechanisms important for the understanding of the effects of autophagy inhibitors on macroautophagy.

The initiation phase of autophagy is preceded by the inhibition of mTORC1. mTORC1 is inhibited by cellular and environmental stresses that are incompatible with continued growth, such as glucose or amino acid deprivation, DNA damage, or hypoxia. mTORC1 consists of three core components: mTOR (highly conserved serine/threonine-protein kinase belonging to the PI3K-related kinase family), RAPTOR (regulatory protein associated with mTOR responsible for mTORC1 localization and substrate recruitment), and mLST8. In addition to these three core components, mTORC1 also contains two inhibitory subunits DEPTOR (DEP domain-containing mTOR interacting protein) and PRAS40 (proline-rich Akt substrate of 40 kDa) [17]. A decrease in cellular energy activates the stress-responsive metabolic regulator AMPK (AMP-activated protein kinase), which inhibits mTORC1 indirectly through activation of Tuberous Sclerosis Complex (TSC), or directly through the phosphorylation of RAPTOR by protein kinase A (PKA) [18, 19]. TSC suppresses mTORC1 by converting Rheb GTPase from an active GTP-bound form to an inactive GDP-bound state. The TSC complex requires G3BPs (Ras GTPase-activating protein-binding proteins) as its lysosomal tether [20].

**Fig. 2:**
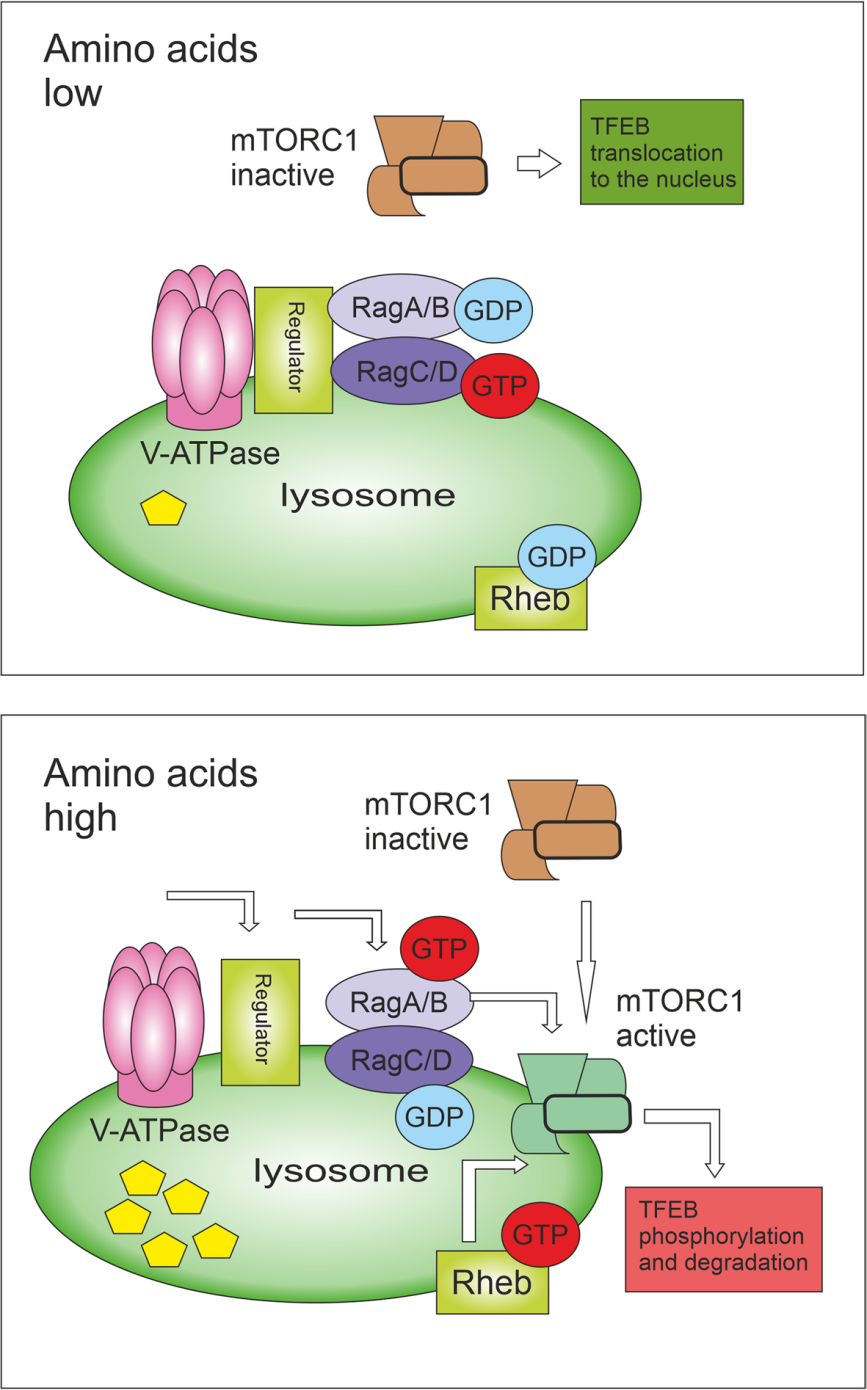
Amino acid-based mTORC1 activation. By amino acid starvation, the inactive V-ATPase-Ragulator complex is unable to activate Rag GTPases on the lysosomal surface, thus mTORC1 is not recruited to the lysosome. The inactivation of mTORC1 leads to rapid translocation of transcription factors TFEB and TFE3 to the nucleus. Active TFEB upregulates the expression of lysosomal genes and critical regulators of autophagy. By amino acid abundancy, the V-ATPase undergoes conformational changes leading to the activation of Regulator, which in turn promotes the Rag heterodimer activation. Active Rag heterodimer (RagA/B(GTP)-RagC/D(GDP)) then recruits mTORC1 to the lysosomal surface where Rheb is present. Rheb can directly bind and activate mTORC1. TFEB is recruited on the lysosomal membrane, phosphorylated by active mTORC1, and then degraded by the proteasome

For sensing the levels of nutrients, the presence of mTORC1 on lysosomes is crucial [21]. In response to amino acids, mTORC1 present on lysosomes can be activated by Rag and Rheb guanosine triphosphatases (GTPases) and can trigger anabolic processes [22]. A key player in Rag-mTORC1 activation is the vacuolar H^+^ ATPase (V-ATPase) that couples ATP hydrolysis (peripheral V1 domain) to proton translocation through the lysosomal membrane (integral V0 domain) to acidify the lysosomes and enable their degradative functions. When the level of amino acids in the lumen of lysosomes is low, the V-ATPase turns off the activity of Rag GTPases. In contrast, when amino acids are abundant, the V-ATPase undergoes conformational changes leading to the activation of Rag heterodimers and the recruitment of mTORC1 to lysosomes [23] (see Fig. 2). Lysosomes are usually localized closer to the plasma membrane when amino acids and growth factors are abundant. On the contrary, when they are limited, the Rap1-GTPases imprison lysosomes in the perinuclear region and reduce lysosome abundance, therefore reducing the lysosomal surface available for mTORC1 activation, which suppresses mTORC1 signalling [24]. The inactivation of mTORC1 leads to rapid translocation of transcription factors TFEB and TFE3 to the nucleus. Active TFEB upregulates the expression of lysosomal genes and critical regulators of autophagy, including several proteins implicated in the formation of autophagosomes and autolysosomes. Therefore, TFEB contributes to the synchronization of autophagy and lysosomes [25]. TFEB can also mediate lysosomal exocytosis and secretion of their cargo including the proteolytic enzymes, such as cathepsins, which results in extracellular matrix remodelling and invasion of cancer cells [26].

**Fig. 3:**
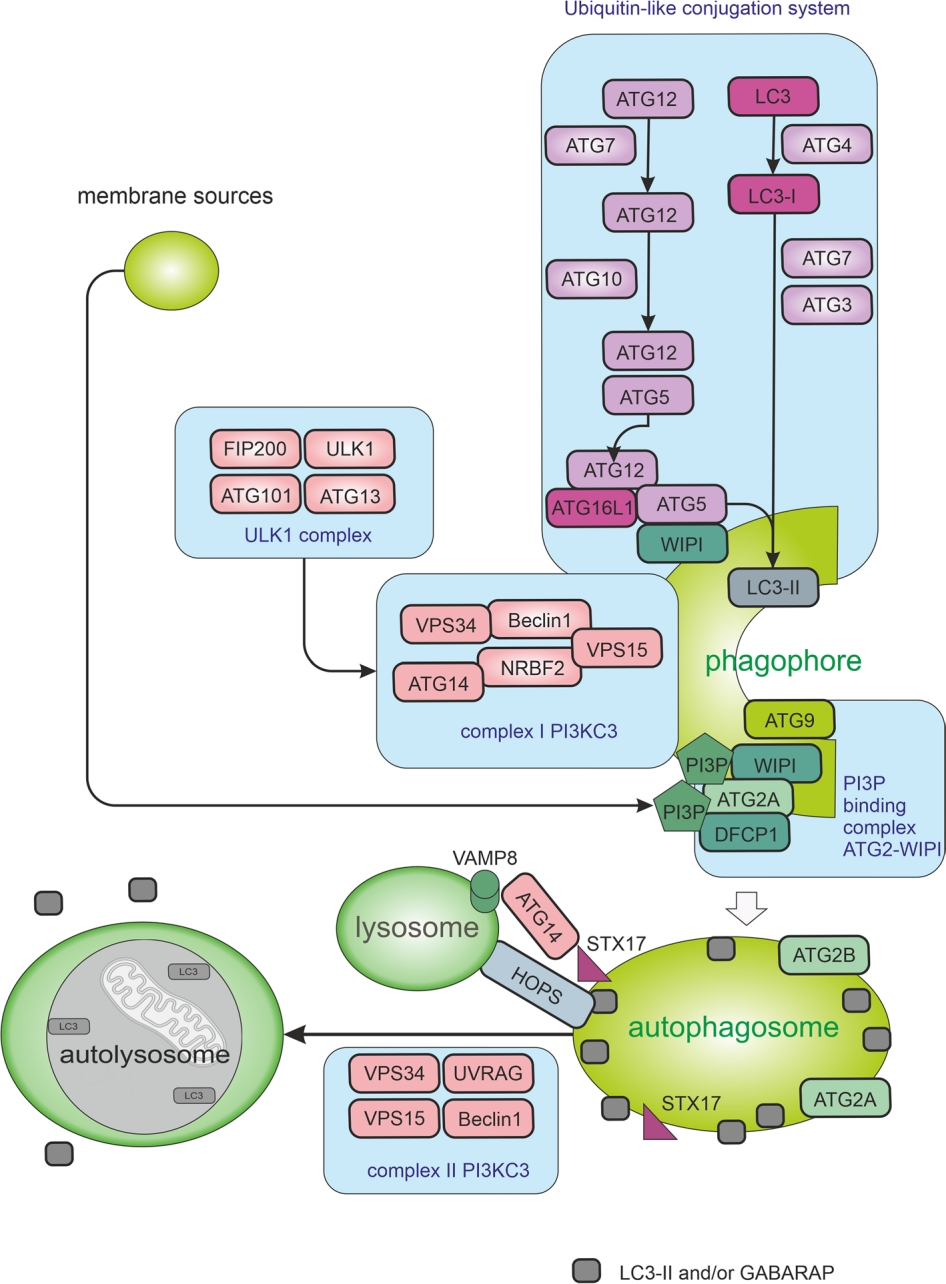
Macroautophagy pathways. The autophagic process is divided into five stages including initiation, phagophore nucleation, phagophore formation, autophagosome-lysosome fusion, and cargo degradation in autolysosomes. Signals activating macroautophagy usually originate from starvation, hypoxia, oxidative stress, and stress of the endoplasmic reticulum (ER). These signals trigger the activity of Unc-51-like kinase 1 (ULK1) complex (consisting of ULK1, FIP200, ATG13, and ATG101), which then starts phosphorylation of components of the class III PI3K (PI3KC3) complex I (consisting of VPS34, VPS15, Beclin1, ATG14L, and NRBF2) enabling nucleation of the phagophore. VPS34 produces phosphatidylinositol-3-phosphate (PI3P) allowing the recruitment of autophagy-associated PI3P-binding proteins such as DFCP1 and WIPI mediating the initial stages of autophagosome formation by associating ATG2A stably to PI3P-containing areas. Expansion of the phagophore requires the ATG2A-WIPI complex mediating ER–phagophore association and establishing the transfer of lipid membranes from the ER and the vesicles to the phagophore. WIPI was also shown to bind ATG16L1, thus recruiting the ATG12–ATG5–ATG16L1 complex. Elongation of autophagosomes requires the ubiquitin-like conjugation system managing the orchestrated activity of ATG proteins and LC3 (microtubule-associated protein light chain 3) and/or GABARAP. The ATG12–ATG5–ATG16L1 complex enhances the final connection of phosphatidylethanolamine (PE) molecules resulting in the formation of membrane-bound LC3-II and/or GABARAP-PE. Cellular membranes, including the mitochondrial membrane, the plasma membrane, recycling endosomes, and the Golgi complex, contribute to the elongation of the phagophore by providing membrane material. Elongation of the phagophore gives rise to double-layered vesicles called autophagosomes. In addition to managing autophagy induction in complex I, complex VPS34-Beclin1 has also a role in the fusion of autophagosomes with lysosomes as complex II. UVRAG competes with ATG14L for binding to Beclin1. When bound to Beclin1, UVRAG stimulates RAB7 GTPase activity and autophagosome fusion with lysosomes. Autophagosome-lysosome fusion is managed by Syntaxin-17 (STX17) on autophagosomes, VAMP8 on lysosomes, and by accessory proteins such as ATG14 and homotypic fusion, and protein sorting (HOPS) tethering complex

The early stage of autophagy machinery is the activation of the ULK1 (unc51-like autophagy-activating kinase 1) complex. This complex consists of ULK1, FIP200, ATG13, and ATG101 (see Fig. 3). ULK1 complex forms puncta usually associated with the endoplasmic reticulum (ER). ER membrane provides local support for many (putatively different) membrane sources and initiates the formation of the isolation membrane (commonly referred to as the phagophore) from the omegasome (a phosphatidylinositide-3-phosphate (PI3P)-enriched subdomain of the ER membrane) [27, 28]. The omegasome serves as a cornerstone for the formation of the phagophore. To the omegasomes, ATG9 vesicles and coat protein complex II (COPII) vesicles are recruited to elongate the autophagosome. The origin of the rest of the lipid bilayers is currently unknown [29]. ATG9 migrates through the trans-Golgi network and the endosomal system under nutrient-rich conditions and transiently binds to the autophagosome in case of autophagy induction. The trafficking of ATG9 through the recycling endosomes may be a fundamental step for autophagosome genesis [30] as ATG9 meets and fuses with ATG16L1 in a VAMP3-dependent manner in recycling endosomes [31].

**Fig. 4:**
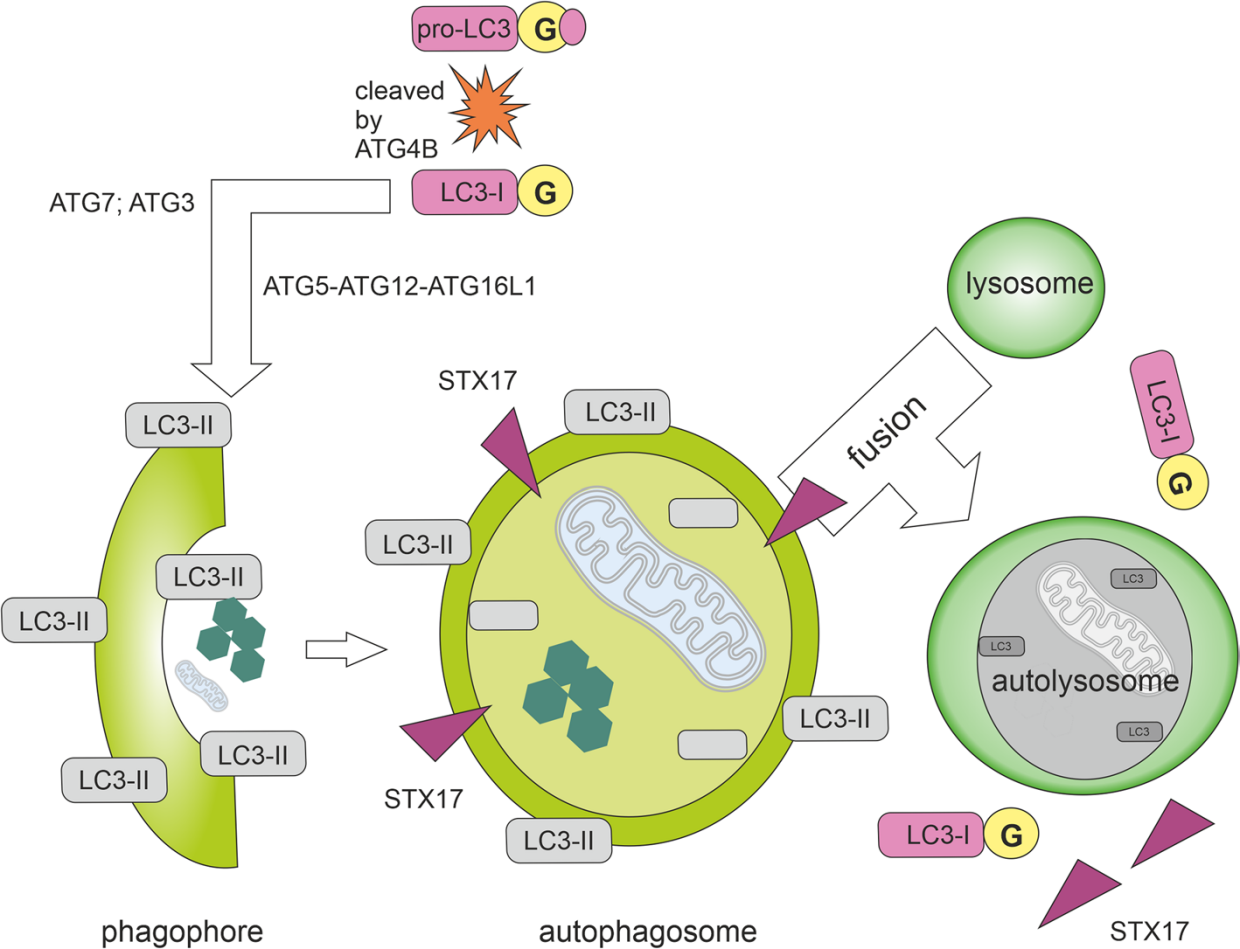
Autophagy with emphasis on the state of MAP1LC3B (LC3B). Newly translated LC3B, called pro-LC3, is cleaved by the cysteine protease ATG4B at the C-terminal end with subsequent exposure of glycine residues (G). A cleaved form of a protein (LC3-I; soluble LC3) is further processed by ATG7 and ATG3, which conjugates LC3-I to phosphatidylethanolamine (PE) molecules. The ATG12–ATG5–ATG16L1 complex enhances the final conjugation of LC3-I to PE molecules resulting in the formation of membrane-bound LC3-II specifically targeted to the elongating autophagosomal membrane. The two ends of the insulating membrane are subsequently joined together to form an autophagosome with a double membrane. STX17, which is located on the outer autophagosomal membrane (not on the isolation or lysosomal membrane), is required for the fusion of an autophagosome with a lysosome. In the resulting autolysosome, the material is cleaved by acid hydrolases. During internal degradation, STX17 proteins are released from the outer membrane of the autophagosome. LC3-II molecules conjugated to the inner autophagosomal membrane are degraded by acids hydrolases, while the LC3-II molecules on the outer membrane of the autophagosome are cleaved by ATG4B and recycled as LC3-I

Once activated, ULK1 phosphorylates the class III phosphoinositide 3-kinase (PI3K) complex I (consisting of VPS34, VPS15, Beclin1, ATG14L, and NRBF2 [16]; see Fig. 3). VPS34 generates PI3P enabling the recruitment of autophagy-related PI3P-binding proteins such as WIPI and DFCP1 [32]. In some circumstances, such as shear stress, PI3KC2α-dependent and VPS34-independent generation of PI3P can take place [33]. Expansion of the phagophore requires the ATG2A-WIPI4 complex mediating ER–phagophore association and establishing the transfer of lipid membranes from the ER and the vesicles to the phagophore [34, 35]. One of the key molecular events of autophagosome formation is the lipidation of ATG8-family proteins with phosphatidylethanolamine (PE). Mammals express 2 subfamilies of ATG8 proteins: the LC3 subfamily consisting of LC3A, LC3B, LC3B2, and LC3C (referred to here as LC3; microtubule-associated protein light chain 3) and the GABARAP subfamily consisting of GABARAP, GABARAPL1, and GABARAPL2 (referred to here as GABARAP). Lipidation of LC3 and GABARAP is a membrane-curvature dependent process [36] catalysed by E1-like activating enzyme ATG7, E2-like conjugating enzyme ATG3 and enhanced by the ATG12–ATG5–ATG16L1 system formed in the previous step [37, 38]. The cysteine protease ATG4B executes two LC3/GABARAP processing events: priming of newly synthesized pro-LC3/GABARAP to enable lipidation (newly translated LC3B, called pro-LC3, is cleaved by the cysteine protease ATG4B at the C-terminal section to give LC3-I) and deconjugation of lipidated LC3/GABARAP after cargo degradation in autolysosome [39] (see Fig. 4). ATG4B is considered to be the main isoform of ATG4 as it possessed the broadest spectrum against all substrates, followed by ATG4A, whereas ATG4C and ATG4D had minimal activity [40]. The final conjugation of LC3-I to PE molecules results in the formation of membrane-bound LC3-II. LC3-II is specifically targeted to the elongating phagophore and remains on autophagosomes until their fusion with lysosomes [37]. The proper closure of the autophagosomal membrane requires the ESCRT-III component CHMP2A and the activity of VPS34 [41]. While the LC3 subfamily mediates the elongation of the phagophore, GABARAP proteins probably function in the final sealing of the autophagosome [42]. GABARAP subfamily positively regulates ULK1 activity and phagophore formation in response to starvation. On the other hand, the LC3 subfamily regulates them negatively [43].

Autophagosomes are transported along microtubules, which require the function of dynein. Consequently, inhibition of dynein-dependent transport or depolymerization of microtubules results in the inhibition of autophagy [44]. The degradative autophagy culminates in a fusion of closed autophagosomes with lysosomes where the cargo is eventually degraded. The autophagosome-lysosome fusion is managed by Syntaxin-17 (STX17) on autophagosomes (see Fig. 4), which binds the VAMP8 (vesicle-associated membrane protein 8) on the lysosomal membrane via the Qbc-SNARE SNAP29 (synaptosome-associated protein 29). Accessory proteins such as ATG14 and homotypic fusion, protein sorting (HOPS) tethering complex, ESCRT, RAB7, and the class C VPS proteins are also needed [45, 46]. Some studies indicate that the kinase ULK1 regulates STX17 engagement during autophagosome maturation. Unphosphorylated ULK1 recruits STX17 and increases its affinity towards SNAP29. Protein kinase C alpha (PKCα) mediated phosphorylation of ULK1 does not change its kinase activity but decreases autophagosome-lysosome fusion [47, 48]. Inactivation of mTORC1 might also be required to facilitate the fusion between autophagosomes and lysosomes [49]. In addition to managing autophagy induction in complex I, complex VPS34-Beclin1 has a role in the fusion of autophagosomes with lysosomes as complex II (see Fig. 3). UVRAG competes with ATG14L for binding to Beclin1 [50]. When bound to Beclin1, UVRAG stimulates RAB7 GTPase activity and autophagosome fusion with lysosomes or multivesicular bodies (late endosomes; MVBs) [51]. VPS34–Beclin1-UVRAG complex may also contribute to autophagy induction via Bif-1/Endophilin B1-mediated activation of VPS34 [52].

## Autophagy in the progression and therapy of solid cancers

Autophagy is undoubtedly an important tumour suppressive mechanism maintaining cellular homeostasis by executing lysosomal degradation of toxic material in the cell, as well as mediating intercellular communication via proteins and hormones with signalling function that can be secreted in an autophagy-mediated manner [53]. During the early phases of cancerogenesis, autophagy has significant cytoprotective and tumour-suppressive potential. Dysfunction of this process is associated with an increased risk of cancer development. Haploinsufficiency of the *BECN1* gene was observed in 40–75% of sporadic human breast and ovarian cancers [54, 55] and more than 25% of gastric and colorectal tumours are haploinsufficient in one of the *ATG2B*, *ATG5*, *ATG9B,* or A*TG12* genes [56]. In multiple cancer types, *ATG5* mutations and alternative mRNA splicing disrupt the ATG16L1-binding to ATG5 and impair the ATG12-ATG5 conjugation. Furthermore, ATG16L2 is overexpressed in several cancers and competes with ATG16L1 for binding to ATG5 resulting in proteasomal degradation of ATG16L1 and disruption of autophagy [57]. The tumour-suppressive effect of autophagy is also supported by the fact that autophagy is stimulated by some tumour suppressors, including PTEN, TSC, or DEPTOR [58–61] (Role of autophagy-related proteins in solid cancers is summarized in Table 1). Nevertheless, if tumourigenesis has been started up, autophagy can further support tumour progression. Many aggressive tumours need autophagy for important tumour-promoting processes (e.g. autophagy enables ERBB2 (Erb-B2 Receptor Tyrosine Kinase 2) trafficking and supports tumourigenesis in ERBB2-driven breast cancer [111]). Increased autophagic activity mediates an escape of premalignant cells from genotoxic stress or anoikis, suppresses immune surveillance and can result in intrinsic resistance against anticancer therapy [112–114]. Autophagy also increases the metabolic plasticity of tumour cells, allowing them to survive in adverse conditions and supports forming of cancer stem cells [115].

**Table 1:** Autophagy-related proteins in cancer

Autophagy-related protein	Type of aberration	Effect on	Type of solid cancer	Reference
AMPK	genetic and transcriptional aberrations	energy homeostasis; tissue-dependent pro- or anticancer impact	many cancer types	[62]
ATG101	overexpression	immunotherapy response	many cancer types	[63]
ATG16L2	overexpression	proteasomal degradation of ATG16L1	many cancer types	[57]
ATG2B, ATG5, ATG9B, ATG12	genetic aberrations, haploinsufficiency	cytoprotection	gastric and colorectal tumours	[56]
ATG9A	overexpression	proliferation	breast cancer	[64]
DEPTOR	reduced expression/overexpression	epithelial to mesenchymal transition (EMT)	low DEPTOR levels in cancer of pancreas, prostate, lungs, triple-negative and breast cancer; high DEPTOR levels in osteosarcoma and differentiated thyroid carcinoma	[60, 65–69]
FIP200	aberrant activation	immune checkpoint therapy response	breast cancer; glioblastoma	[70, 71]
mLST8	overexpression	cancer progression	hepatocellular carcinoma	[72]
mTORC1	overactivation	survival of stem cells; reprogramming of metabolism; tumour invasion and metastasis	many cancer types	[73–75]
PRAS40	overexpression	proliferation; enhanced NF-κB activity	hepatocellular carcinoma; lung adenocarcinoma; cutaneous melanoma	[76, 77]
RAP1	changes in the RAP1 activation	formation of cell adhesions and junctions; migration and polarization; may suppress oncogenic Ras phenotype	Rap1 inhibits invasion and metastasis in the bladder, lung, and brain cancer; it has the opposite effect in melanoma and breast cancer or oesophageal squamous cell, head and neck squamous cell pancreatic, and non-small cell lung carcinomas	[78, 79]
RAPTOR	overexpression	proliferation and migration; the resistance to PI3K-mTOR inhibition	colorectal cancer; renal cancer; oropharyngeal squamous cell carcinoma	[80–82]
TFE3	gene fusions	insulin-dependent metabolism; retinoblastoma-dependent cell cycle arrest	renal cell carcinoma	[83]
TFEB	gene fusions, transcriptional aberrations	biology of lysosomes; lysosomal exocytosis; proliferation; glutamine metabolism; regulator of tumour-associated macrophages; role in the TME; WNT and TGFβ signalling	Pancreatic, breast , and renal cancer; melanoma; colorectal cancer; gastric carcinoma; non-small cell lung cancer	[84–87]
TSC1/2	genetic aberrations	anticancer impact	many cancer types	[88]
ULK1	aberrant activation, genetic and transcriptional aberrations	antitumour immunity; NADPH production; innate immune response; cell cycle progression	LKB1-mutant lung cancer and many other types of cancer	[89–92]
V-ATPase	deregulation of some subunits, overexpression	biogenesis of endosomes and lysosomes; treatment resistance	breast cancer, lung and oesophagal tumours	[93–95]
VPS34	aberrant activation	antitumour immunity; survival of cancer stem cells; activation of p62; antigen cross-presenting CD8α+ dendritic cells	melanoma; colorectal cancer; hepatocellular carcinoma	[96–99]
WIPI3	genetic and transcriptional aberrations	cell cycle and spliceosome	hepatocellular carcinoma	[100]
VAMP3	deletion of WDFY2 (negative regulator of VAMP3 recycling)	increase in extracellular matrix degradation	metastatic ovarian and prostate cancers	[101, 102]
GABARAP	transcriptional aberrations	tumour differentiation; EMT	colorectal cancer; breast cancer	[103, 104]
MAP1LC3A or B	transcriptional aberrations	cancer progression	breast cancer; renal cell carcinoma	[105, 106]
p62/SQSTM1	accumulation	cancer progression	many cancer types	[107]
RAB7	reduced expression	biogenesis of endosomes, autophagosomes, and lysosomes	highly invasive breast cancer	[93]
ESCRT	polymorphism in ESCRT-III (rs35094336, CHMP4C)	endosomal-sorting; cytokinetic abscission; genome instability	many cancer types	[108]
UVRAG	inhibition	cytokine production; oncogenic signalling; progression of age-related malignancies	many cancer types	[109]
Beclin1	genetic and transcriptional aberrations	cytoprotection; proliferation under hypoxia and nutrient starvation	haploinsufficiency in breast and ovarian cancer; overexpression in colorectal and gastric carcinomas	[54, 55, 110]

Because the induction of autophagy has been observed as a side effect of many cytotoxic anticancer therapies causing therapy resistance, a large number of strategies using autophagy inhibition have been proposed to increase the efficacy of these therapies [116]. Inhibition of autophagy in the tumour microenvironment can disrupt metabolic communication between tumour and stromal cells [117] and decrease cell motility of metastatic tumour cells as inhibition of autophagy reduces disassembly of focal adhesions at the leading edge of the cell [118]. Systemic acute deletion of ATG7 in adult mice with preexisting non-small cell lung cancer reversed lung adenocarcinomas initiated by the KRASG12D oncogenic mutation and p53 deficiency to a benign form of tumour (oncocytomas) and blocked cell proliferation and cancer cell survival [119]. Furthermore, loss of tuberous sclerosis complex 2 (TSC2) sensitizes cancer cells to nelfinavir−bortezomib therapy due to intensifying endoplasmic reticulum stress-induced cell death [61]. On the other hand, autophagy induction causes a decrease in the levels of transcription factors triggering EMT [120], reduces 6-phosphofructo-2-kinase/fructose-2,6-biphosphatase 3 (Pfkfb3) expression and elicits metastatic dormancy in breast cancer stem cells [121]. Consequently, many studies are demonstrating the benefit of autophagy during cancer therapies, especially in inducing immunogenic cell death. Tumour cells dying of autophagic cell death leads to the recruitment of immune cells to the tumour site and the activation of a tumour-specific immune response. Accordingly, caloric restriction (which promotes autophagy by inactivating mTORC1) has resulted in increased control of the immune system over the tumour, but only in tumours capable of autophagy [122].

Autophagy may be one of the key modulators of the tumour microenvironment (TME). The autophagy-dependent secreted soluble factors or factors contained in EVs may enable metabolic manipulation of non-cancer cells in the tumour microenvironment, stimulate cellular proliferation, invasive phenotype, and promote immunosuppression [53]. This TME-modulating theory is supported by the fact that autophagy facilitates the selection of material for unconventional secretion of certain cytoplasmic proteins [123]. Intact autophagy machinery is required for the secretion of multiple factors favouring invasion, including interleukin-6 (IL-6), MMP2, and WNT5a [124]. Autophagy is also closely related to the biogenesis and secretion of exosomes and amphisomes.

## Autophagy and MVBs

Autophagy and endocytic pathways are important in managing many aspects of homeostasis as both endosomes and autophagosomes are known to deliver cellular material to lysosomes for degradation. Autophagy and exocytosis seem to be largely interconnected as autophagy cargo can be released by amphisomes derived from multivesicular bodies (MVBs) and phosphoinositide-3-phosphate (PI3P) is essential for the genesis of both endosomes and autophagosomes, and their positioning as PI3P promotes the microtubule-dependent translocation of late endosomes and lysosomes to the cell periphery. This PI3P-dependent lysosome translocation to the cell periphery promotes mTORC1 activation [125]. The most of cellular PI3P is generated by class III PI3K VPS34 in complex II with a small contribution of class II PI3Ks [126]. Binding and activation of VPS34 on endosomes are initiated through the recruitment of RAB5 to endosomes by the guanine nucleotide exchange factor Rabex5 (RAB Guanine Nucleotide Exchange Factor 1) [127]. VPS34 then produces PI3P increasing the binding of RAB5 and other downstream effectors, including early endosome autoantigen 1 (EEA1), the hepatocyte growth factor-regulated tyrosine kinase substrate (HRS; ESCRT-0 subunit) regulating MVBs formation via ESCRT recruitment to endosomes [128]), and endosomal sorting nexin protein family (SNX) [129]. Overexpression of SNX3 may alter the morphology of endosomes and delay their transport to the lysosome [130]. SNX18 was identified as a positive regulator of autophagosome formation [131]. Consequently, VPS34 plays a crucial role in endosome biogenesis through EAA1 and other RAB5 effectors, vesicle invagination and cargo selection within MVBs, and the fusion of autophagosomes with lysosomes. Inhibition of VPS34 resulted in dysfunction in autophagy, vesicular trafficking, and endocytic recycling and sorting [132, 133]. Furthermore, proteins such as Beclin1 and ATG14L that regulate PI3P levels are positive modulators of autophagy [134]. Some data suggest that surface delivery of endosomal cargo requires hydrolysis of PI3P mediated by MTM1 as the endosomal accumulation of PI3P inhibits exocytosis. Defects caused by mutations in MTM1 can be partially reversed by pharmacological inhibition of VPS34 [135].

**Fig. 5:**
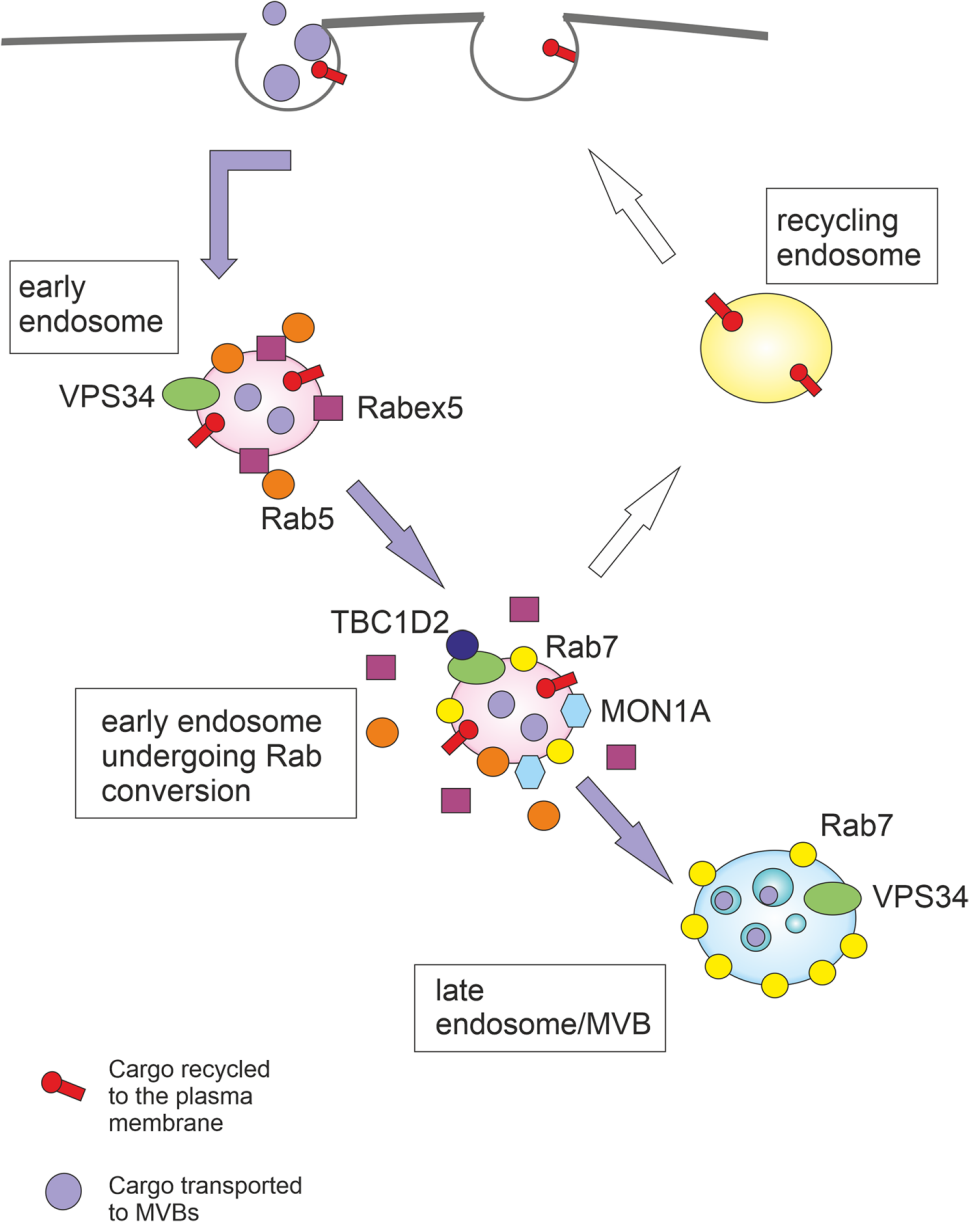
The transition from early to late endosomes. Binding and activation of VPS34 on endosomes are initiated through the recruitment of RAB5 to endosomes by the guanine nucleotide exchange factor Rabex5. VPS34 then produces PI3P increasing the binding of RAB5 and other downstream effectors. The transition from early to late endosomes is complicated by a positive feedback loop between Rabex5 and RAB5. MON1A is needed to interrupt this positive feedback loop by displacing Rabex5 from endosomal membranes. MON1A also manages the recruitment of RAB7. VPS34 recruits TBC1D2 protein to endosomes in a RAB7-dependent manner to further inactivate RAB5 and to facilitate early to late endosome maturation

Endosomal maturation is accompanied by conversion from early endosomal RAB5 to late endosomal RAB7 and active cargo sorting into intraluminal vesicles (ILVs) by the ESCRT complex. The transition from early to late endosomes is complicated by a positive feedback loop between Rabex5 and RAB5. It was demonstrated that SAND1/MON1A is needed to interrupt this positive feedback loop by displacing Rabex5 from endosomal membranes. SAND1/MON1A also manages the recruitment of RAB7 (see Fig. 5) [127]. Then VPS34 recruits TBC1D2 protein to endosomes in a RAB7-dependent manner to further inactivate RAB5 and to facilitate early to late endosome maturation. VPS34 inhibition causes hyperactivation of RAB7, autolysosomal dysfunction, a phenotype with large late endosomes and an enhanced release of atypical exosomes harbouring poly-ubiquitinated proteins [136–138]. Interestingly, RAB7 can participate in both MVBs degradation and/or MVBs-related secretion as it regulates autolysosome maturation and simultaneously the secretion of syntenin and syndecan-containing exosomes [139]. RAB7-associated endosomal processes depend not only on RAB7 GTP-based state but also on modifications with ubiquitin [140]. Endosomal maturation during the late endosome/lysosome pathway is accompanied by conversion of PI3P to PI(3,5)P_2_ at the limiting membrane of late endosomes. This process depends on PIKfyve (phosphoinositide kinase, FYVE-type zinc finger containing). Consequently, the activity of PIKfyve is vital for the sorting of cargo into MVBs [141].

**Fig. 6:**
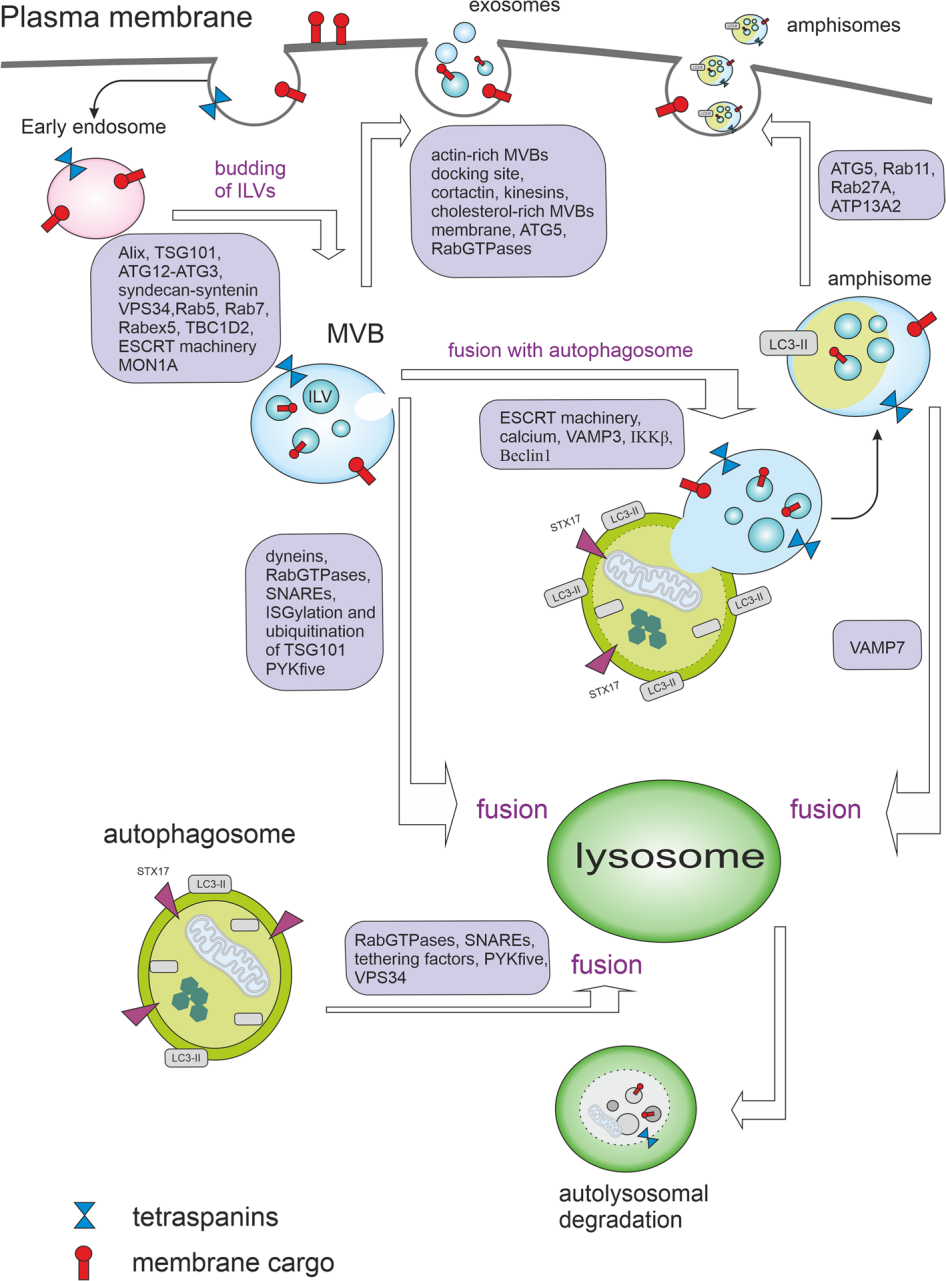
Multivesicular bodies and autophagy. After maturation of early endosomes to multivesicular bodies (MVBs), MVBs can fuse with the plasma membrane to release intraluminal vesicles (ILVs) to the extracellular space as exosomes. With the help of specific proteins, MVBs are trafficked towards the plasma membrane and/or lysosome. Under certain conditions, MVBs can fuse with autophagosomes to generate hybrid organelles called amphisomes. Amphisomes contain typical autophagosomal markers such as lipidated LC3, and due to their origin from endosomes, they also contain endosomal markers such as CD63, RAB5, RAB7, and RAB11. The fusion of MVBs with the lysosome (direct or via autophagosome) results in autophagic degradation. The MVBs-related secretion and autophagy pathways are connected via many proteins, including RAB GTPases, ESCRTs, SNAREs, Beclin1, ATG proteins, and LC3

In addition to the role in the degradation and recycling of cellular waste, autophagic and endo-lysosomal systems can play a key role in secretory pathways (see Fig. 6) as autophagy cargo can be released by amphisomes derived from multivesicular bodies (MVBs). MVBs are late endosomes containing many intraluminal vesicles (ILVs) formed by the invagination of the endosomal membrane. ILVs start to be generated in early endosomes and accumulate until the late endosomal stage. *In vitro* budding of ILVs into MVBs is regulated by syntenin-syndecan interactions requiring Alix which is known to interact with several ESCRT proteins including TSG101 and CHMP4 [142, 143]. MVBs can fuse with the plasma membrane to release intraluminal vesicles (ILVs) to the extracellular space as exosomes. During exosome biogenesis, Alix forms a complex with the scaffold protein syntenin, mediating the loading of cargo into exosomes and promoting exosome release. These Alix-dependent processes are controlled by ATG12–ATG3 and cells lacking ATG12–ATG3 showed reduced exosome biogenesis. Both ATG12–ATG3 and Alix promote basal, but not starvation-induced, autophagic flux [9]. Some results indicate that activated c-Src in the endosomal membrane promotes the secretion of exosomes. Alix was identified as a c-Src–interacting protein in exosomes, resulting in the upregulation of exosome secretion in c-Src–transformed cells [143]. The small GTPase ADP ribosylation factor 6 (Arf6) and its effector phospholipase D2 (PLD2) also regulate the syntenin pathway [144]. MVBs morphology and their docking to the plasma membrane is significantly disrupted by the loss of RAB27a and/or RAB27b activity [145]. Critical plasma membrane docking and secretion sites for MVBs are invasive actin structures called invadopodia. Invadopodia degrade the extracellular matrix through the local deposition of matrix metalloproteinases (MMPs) and manage cancer cell invasion [146]. Protein WDFY2 (WD Repeat and FYVE Domain Containing 2) is frequently lost in metastatic cancers (e.g. ovarian and prostate cancers). Through its interaction with VAMP3, WDFY2 restricts the budding of MMP14-containing VAMP3 vesicles from actin-stabilized endosomal tubules. Upon deletion of WDFY2, this negative control is disrupted and faster recycling of MMP14 to the plasma membrane leads to increased matrix degradation and cell invasion [102]. Furthermore, long non-coding RNA HOTAIR promotes the colocalization of VAMP3 with SNAP23 leading to MVBs fusion with the plasma membrane and exosome secretion in hepatocellular carcinoma [147]. The HOTAIR also activates autophagy by upregulating ATG3 and ATG7 [148]. Long intergenic noncoding RNA 00511 (LINC00511) promotes exosome secretion by regulation of the expression of RAB27B and the colocalization of VAMP7 and SNAP23, which are involved in MVBs trafficking and their fusion with the plasma membrane [149]. LINC00511 is highly expressed in diverse cancers and correlates with poor clinical outcomes [150]. LINC00511 knockdown suppressed proliferation, invasion and autophagy in trophoblast cells [151]. Exosome secretion was also promoted by cortactin through stabilizing cortical actin-rich MVBs docking sites [152].

Nevertheless, in healthy cells, the majority of MVBs fuse with the lysosomes, resulting in degradation of their content. ISGylation of the MVB protein TSG101 by ISG15 (interferon-α/β-induced ubiquitin-like protein) induces its aggregation and degradation, being sufficient to impair exosome secretion. The secretion of exosomes is recovered when the fusion of MVBs with lysosomes or autophagosomes is inhibited, indicating that the inhibition of exosome secretion is mainly mediated by the induction of MVBs degradation by the lysosomes. ISGylation reduces the number of MVBs but does not inhibit the formation of ILVs. It also promotes selective autophagy and degradation of MVBs without inducing a global autophagy response [153]. The fusion of MVBs with lysosomes can be also triggered via the ubiquitination of TSG101 by MGRN1 [154].

Interestingly, ubiquitination of the cargo proteins can also profoundly influence the fate of MVBs. While MVBs containing ubiquitinated major histocompatibility complex (MHC-II) underwent lysosomal degradation in immature dendritic cells, non-ubiquitinated MHC-II placed into CD9-bearing MVBs was targeted for plasma membrane fusion and secretion in activated dendritic cells. Sorting of MHC II into exosomes correlated with its incorporation into CD9 containing detergent-resistant membranes [155]. Exosomes are probably preferentially secreted from cholesterol-rich detergent-resistant MVBs whereas the cholesterol-poor MVBs are subjected to degradation [156, 157]. The alternative fate of MVBs can be also governed by their connection to dynein or kinesin. Connection to dynein motor protein (minus end-directed transport) leads to perinuclear accumulation of MVBs and lysosomal degradation of cargo by recruitment of HOPS complex. By contrast, connection to kinesin (plus end-directed transport) causes accumulation of MVBs at cell periphery [158, 159].

Different autophagic effectors were shown to influence the biogenesis of extracellular vesicles and their secretion. Exosome production is strongly reduced in cells lacking ATG5 and ATG16L1 as these proteins protect MVBs from lysosomal degradation and direct them into the secretory pathway. ATG5 detaches V1V0-ATPase (vacuolar proton pump) from the MVBs via LC3 which specifically decreases acidification of MVBs lumen [160]. Emerging exosomes were strongly enriched in LC3-II (versus LC3-I) compared with the ratio of LC3-II to LC3-I in the corresponding cell lysates. This suggests that ATG5 and LC3 are sorted into these exosomes [160]. On the other hand, overexpression of LC3 or autophagy inducers such as starvation or rapamycin caused an enlargement of the vacuoles decorated with RAB11 and their colocalization with LC3. This situation led to the inhibition of exosome release. Even though RAB11 activity stimulates MVBs genesis and exosome release [161], the stimulatory effect of RAB11 in exosome secretion was nullified by overexpression of the autophagic protein LC3 [10].

Under certain conditions (such as shear stress [162]), autophagosomes fuse with MVBs to generate hybrid organelles called amphisomes [13]. Amphisomes contain typical autophagosomal markers such as lipidated LC3, and due to their origin from endosomes, they also contain endosomal markers such as CD63, RAB5, RAB7, and RAB11 [163]. Maturation of MVBs by ESCRT machinery is required for their fusion with autophagosomes [164] and the fusion of MVBs with the autophagosome compartment seems to be calcium- and VAMP3-dependent event [10, 163]. The resulting amphisomes are either degraded by fusion with lysosomes or released from the cell [162, 165]. ATG9 is required to form intraluminal vesicles in amphisomes and/or autolysosomes and is also needed for local acidification within amphisomes/autolysosomes [166]. The fusion between amphisomes and lysosomes requires VAMP7 but not VAMP3 [163]. VAMP7 is also needed for the homotypic fusion of ATG16L1 precursors, which is a key event in the early phases of autophagy enabling membrane acquisition and autophagosome biogenesis [167]. VAMP7-labelled vesicles can be loaded with ATP and starvation triggers the delivery of the ATP-containing amphisomes toward the plasma membrane, their release, and the import of ATP to the extracellular space [168].

Amphisomes, but not exosomes, were shown to be vehicles for the active release of DNA from the cell [169]. The formation of an amphisome may negatively regulate the coordination between exosome secretion and autophagy. For example, rapamycin or starvation promoted MVBs-autophagosome fusion and reduced exosome secretion in the K562 cell line [10]. On the other hand, the exosome release of toxic/damaged material may provide a cellular mechanism bypassing the autophagic defects caused by ageing or different pathological states [170].

Amphisomes can participate in immune response as they can function as anti-viral machinery by sequestering and exporting viral proteins from the cell [171]. IFNγ is a cytokine critical for innate and adaptive immunity against viral infections. Upon IFNγ-induced autophagy in lung epithelial cells, amphisomes containing annexin 2 (ANXA2) were released. This process was dependent on ATG5, RAB11, and RAB27A [165].

Interleukins IL-1β and IL-18 are potent pro-inflammatory cytokines crucial for responses to infection and injury. Secretion levels of these cytokines increased when low-autophagy cells were treated with the autophagy-inducing tat-Beclin1 peptide and decreased when ATG7 was silenced in high-autophagy cells [172]. An unobstructed autophagy pathway and functional MVBs are necessary for inflammasome-dependent IL-1β and IL-18 secretion [173, 174]. Nevertheless, it is not completely clear if IL-1β is secreted by amphisomes or by modified autophagosomes. Similarly to the degradative autophagosomes, modified secretory autophagosomes have a double membrane decorated with LC3-II. In degradative autophagosomes, STX17 manages the fusion with the lysosome. However, in secretory autophagosomes, SEC22B in combination with plasma membrane syntaxin 3 and syntaxin 4 as well as SNAP23 and SNAP29 facilitate fusion with the plasma membrane and cargo secretion [175, 176]. Autophagy-dependent secretion of IL-1β, IL-6, CSF3/G-CSF, CXCL1, TREM1, CCL2, CCL3/MIP-1α, and CXCL2 in response to UVB radiation was also described. Secretion of these cytokines was blocked by conditional ATG7 depletion [177].

Another factor interconnecting cell stress and immune response with autophagy and amphisome genesis is IκB kinase (IKKβ). IKKβ is the predominant catalytic subunit of the IKK complex and is required for the activation of the canonical NF-κB signalling pathways. IKKβ activation also induces the fusion of MVBs with autophagosomes to form amphisomes and promotes their secretion in tumour cells. This secretion was absent or strongly reduced when autophagosome formation was impaired by 3-methyladenine or ATG7 inactivation [178]. In breast cancer cells independent of autophagy, ATG7 inhibition by shRNAs increased IL-6 secretion. On the other hand, in autophagy-dependent cells, ATG7 inhibition decreased IL-6 secretion, cell survival and mammosphere formation [179]. Besides RNA interference-mediated ATG7 depletion, pyrazolopyrimidine sulfamate compounds were found to be potent selective inhibitors of ATG7 [180].

In cancer cells, selective and non-selective autophagy and EVs secretion are often amplified because of harsh conditions in TME, such as hypoxia or ER stress [181–183]. Some regulators such as GAIP interacting protein C terminus (GIPC) control both EVs and autophagy pathways [184]. GIPC knockdown led to significant inhibition of pancreatic carcinoma growth in an orthotopic mouse model [185]. The crosstalk between endosome-related secretory pathways and autophagy orchestrates the intratumoural communication as autophagy significantly impacts not only the quantity of EVs but also their content. On the other hand, EVs can significantly influence the dynamic of autophagy in TME [186, 187]. Exosomes derived from breast cancer cells stimulate beige/brown differentiation and reprogram metabolism in stromal adipocytes to promote cancer progression [187]. It was also shown that hypoxic glioma-derived exosomes promote M2-like macrophage polarization by enhancing autophagy induction [188] and MCF-7 breast cancer cells with undetectable MMP2 protein acquired expression of MMP2 and corresponding gelatinase activity after stimulation with exosomes derived from mesenchymal stromal stem cells [189].

## Autophagy modulators and their effect on EVs secretion

**Fig. 7:**
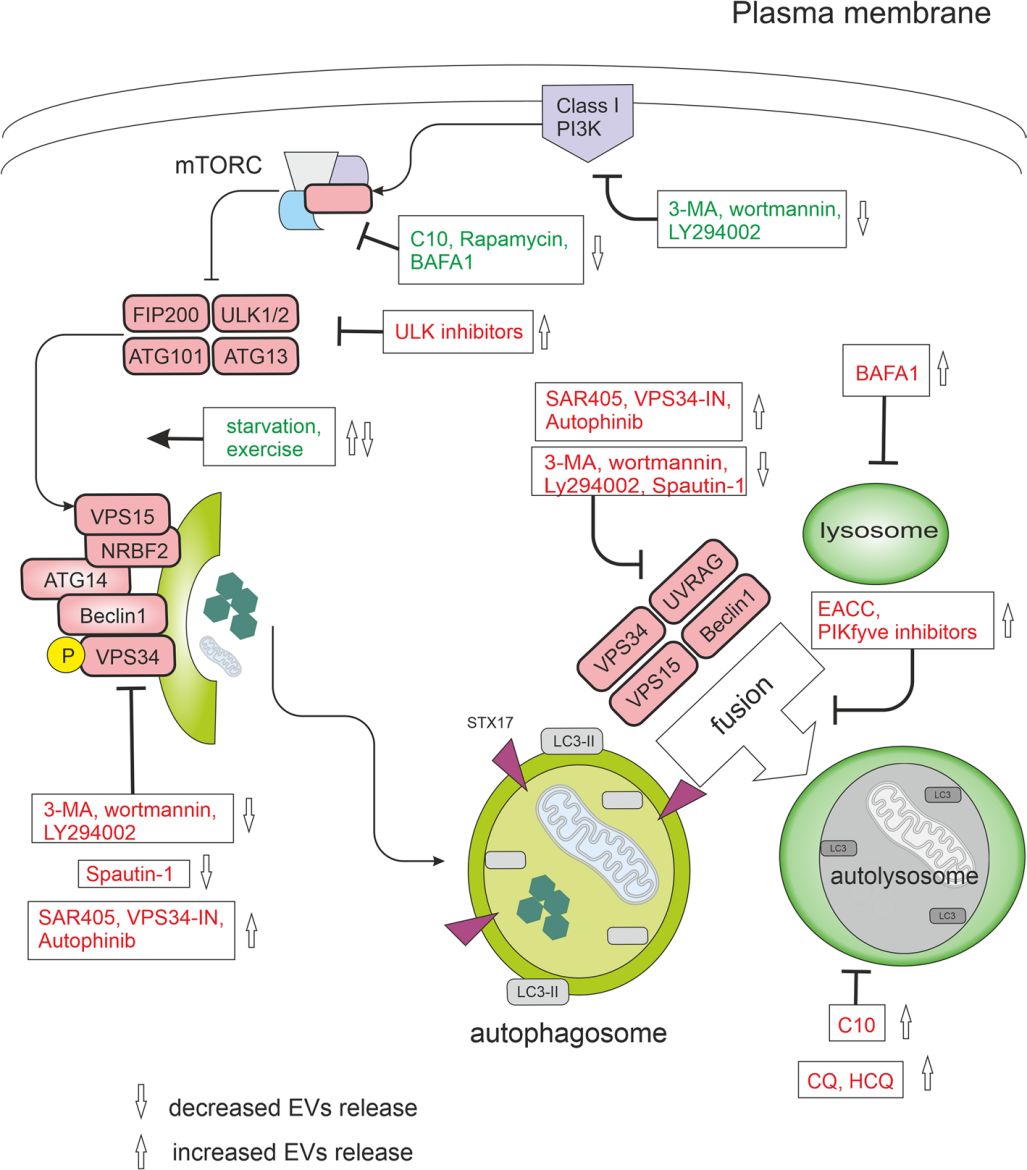
Modulators of autophagy and their effect on EVs release. In nutrient-rich conditions, mTORC1 constitutively blocks the ULK complex and autophagy. mTORC1 signals can be inhibited directly by C10, rapamycin, exercise, or starvation and indirectly by bafilomycin A1 (BAFA1) through lysosomal inhibition. Physical exercise was shown to induce the release of small extracellular vesicles (EVs) into the circulation [195]. The ULK complex activates the VPS34 complex. VPS34 is a class III phosphatidylinositol 3-phosphate-kinase (PI3KC3). A group of PI3K inhibitors, including 3-methyladenine (3-MA), wortmannin, and synthetic inhibitor LY294002, inhibits both class I as well as class III PI3Ks. VPS34 inhibitors include Spautin-1, autophinib, SAR405, and VPS34-IN1. Spautin-1 initiates the degradation of Beclin1 due to the inhibition of two of its deubiquitinases. SAR405 and VPS34-IN1 are highly potent inhibitors of VPS34 selective for the VPS34 and not affecting the closely related class I and class II PI3Ks. Autophinib is an ATP-competitive inhibitor of VPS34 decreasing the accumulation of the lipidated protein LC3 on the autophagosomal membrane. The late stages of the autophagic machinery include fusion and degradation. During fusion, the mature autophagosome fuses with lysosomes creating an autolysosome. PIKfyve (phosphoinositide kinase, FYVE-type zinc finger containing) inhibitors and EACC block autophagosome-lysosome fusion. BAFA1 inhibits the acidification of the autolysosome by blocking the V-ATPase while chloroquine (CQ) and 3-hydroxychloroquine (HCQ) impair the maturation of autolysosomes. All drugs are depicted within the rectangles. Effects of modulators activating autophagy are green, inhibitory effects are red

Autophagy is a multi-step process. Each step can potentially be inhibited. Progress within the field has led to the development of agents targeting almost all phases of this process (see Fig. 7). Targeting the specific stage of autophagy may profoundly influence resulting secretory pathways as the early-stage autophagy inhibition does not seem to be equivalent to the late-stage inhibition. Furthermore, one compound (such as tacrine-melatonin heterodimer C10) can induce the early stages of autophagy and inhibit it at the late stages. Transitory treatment by C10 induced secretion of proinflammatory cytokine IL-6, proving interconnection between autophagy and secretory pathway [190]. Some amount of IL-6 produced was found to be secreted by exosomes [191, 192]. On the other hand, IL-6 inhibits starvation-induced autophagy and activates stress-induced autophagy via the STAT3 signalling pathway [193, 194].

### ULK1 inhibitors

ULK1 is a serine/threonine-protein kinase. In most cell lines, loss of ULK1 is sufficient to disrupt autophagy [196]. However, ULK2 acts with a degree of redundancy with ULK1 [197]. MRT68921 potently inhibits both ULK1 and ULK2. ULK1 inhibition results in the accumulation of immature early autophagosomal structures, indicating a role for ULK1 in the initiation and maturation of autophagosomes [198]. ULK1 activity also manages the trafficking of ATG9 [199] which is required for intraluminal vesicle formation within amphisomes and autolysosomes [166]. Consequently, inhibition of ATG9 causes a reduced capacity to degrade endosomal cargo, which may result in enhanced EVs secretion. Amino acid starvation or rapamycin causes a redistribution of ATG9 from the trans-Golgi network to peripheral, endosomal membranes. The redistribution of ATG9 requires PI3K activity and is reversed after the restoration of amino acids [199]. On the other hand, AMPK-ULK1-mediated but mTOR-independent signalling plays an important role in the induction of autophagy-mediated PARK7 secretion. 6-hydroxydopamine-induced oxidative stress triggered PARK7 secretion which was suppressed by co-treatment with MRT68921 [200]. Nevertheless, PARK7 seems not to be secreted by classical exosomes [169]. MRT68921 can also disrupt the signals between lysosomes and autophagic machinery. The lysosomal calcium channel TRPML1 connects lysosomal calcium to autophagosome biogenesis through the triggering of the CaMKKβ/VPS34 pathway. TRPML1-mediated generation of PI3P requires functional VPS34 and ULK1 [201]. RNAi-mediated knockdown of ULK1 and/or ULK2 resulted in impaired endocytosis of nerve growth factor (NGF) [202]. MRT68921 was also shown to be a potent inhibitor of NUAK1 (NUAK family SNF1-like kinase 1) which is a critical component of the antioxidant defence necessary for the survival of tumour cells during cytotoxic therapy and EMT. As cytotoxic therapy induces elevated ROS levels and triggers the ULK1 pathway to activate protective autophagy and mitophagy, dual targeting of NUAK1 and ULK1 by MRT68921 can be beneficial in tumour management [203]. ULK1 inhibition also overcomes compromised antigen presentation in LKB1 (liver kinase B1)-mutant lung cancer [89].

### Pan-PI3K inhibitors

Phosphoinositide 3-kinases (PI3Ks) are divided into three classes: class I, class II, and class III. Class I PI3Ks predominantly produce PI(3,4,5)P3 (and indirectly, PI(3,4)P2), class III PI3Ks synthesise PI3P, and to a lesser extent, class II PI3Ks synthesise PI3P and PI(3,4)P2). PI(3,4)P2 was described as a key mediator of the late stages of clathrin-mediated endocytosis [126]. PI(3,4,5)P3 at the plasma membrane recruits effectors such as the protein kinase B/Akt. Active Akt controls many downstream pathways such as the mTORC1 pathway. Activated mTORC1 destabilizes the ULK1 complex and inhibits autophagy [204]. The role of PI3P has been discussed above.

A group of PI3K inhibitors includes 3-methyladenine (3-MA), wortmannin, and synthetic inhibitor LY294002. These compounds inhibit class I PI3Ks as well as class III PI3Ks. Wortmannin and 3-MA have been routinely used as autophagy inhibitors due to their suppressive effect on class III PI3Ks. Nevertheless, 3-MA with a prolonged period of treatment (up to 9 hours) was also found to promote autophagy even under nutrient-rich conditions. 3-MA also does not inhibit Beclin1-independent autophagy [205], however, it can still suppress starvation-induced autophagy [206]. In contrast, wortmannin suppresses autophagy regardless of the nutrient status [206]. The effect of 3-MA (but not wortmannin) is further complicated by differential temporal effects on class I and class III PI3Ks. 3-MA persistently blocks class I PI3Ks, whereas its impact on class III PI3Ks is only transient [206]. Furthermore, activation of autophagy was shown in gastric cancer cell line SGC7901 due to LY294002 treatment [207]. Another problem is that LY294002, and to a lesser extent wortmannin (but not 3-MA [208]), can inhibit proteosynthesis [209] and 3-MA can induce a decrease in cell viability not driven by the inhibition of the Akt/mTOR axis. The cytotoxicity induced by 3-MA correlated with massive DNA damage monitored by γ-H2AX and was observed using the 10 mM concentration, the usual concentration used to inhibit autophagy [210].

Off-target activities of PI3K inhibitors influence proteasome degradation, PI3K/Akt signalling pathways, endocytosis, lysosomal acidification, mitochondrial permeability transition, and glycogen metabolism [211, 212]. Moreover, it is known that PI3Ks also participate in the biogenesis of MVBs and their activity is required for the correct maturation of the ILVs [10, 129]. As would be expected, factors having some effect on the formation of MVBs also affect the secretion of exosomes. Consequently, conventional exosome production can be decreased by PI3K inhibitors [213–215]. Accordingly, wortmannin reduced the secretion of prostasomes from PC-3 cells [216]. On the other hand, inhibition of autophagy by wortmannin or CRISPR/Cas9-mediated knockout of ATG5 greatly increased the release of exosome-associated prions [217]. In fibroblasts, wortmannin caused swollen endosome phenotype coupled with the failure of membrane recycling but not the inhibition of MVBs biogenesis [218]. Nevertheless, wortmannin can change the inner content of ILVs [129]. The efficacy of wortmannin may be diminished by its covalent binding to free amino acids [219].

Inhibition of autophagy with 3-MA or wortmannin can have profound effects on cytokine secretion. In macrophages, toll-like receptor ligands initiate sequestration of pro-IL-1β into autophagosomes and activation of autophagy with rapamycin triggered the degradation of this sequestrated pro-IL-1β and blocked secretion of the mature cytokine. When treated with 3-MA or wortmannin, LPS-stimulated bone marrow-derived macrophages (iBMM) and dendritic cells (BMDC) secreted increased levels of IL-1β. At the 10 mM concentration, 3-MA induced IL-1β but inhibited IL-6 secretion from BMDC and iBMM. In contrast, 3-MA and wortmannin markedly reduced IL-1β secretion induced by LPS + ATP in human neutrophils [220]. 3-MA, in combination with LPS, increased IL-1α secretion by BMDC and IL-18 secretion by iBMM. 3-MA also activates the inflammasome through inhibition of type III PI3Ks [221]. Wortmannin was found to enhance IL-12 production in dendritic cells [222]. In contrast, LY294002 prevented IL-12 secretion in dendritic cells [209].

### VPS34 inhibitors

VPS34 is involved not only in autophagy but also in the endosomal trafficking of receptors such as the epidermal growth factor receptor (EGFR) [129]. In addition to managing autophagy induction in complex I, VPS34 also has a role in the fusion of autophagosomes with lysosomes. Disruption of neuronal VPS34 function impairs autophagy, lysosomal degradation, as well as lipid metabolism, causing endolysosomal membrane damage. PI3P deficiency caused by a malfunction of VPS34 also promotes the secretion of unique exosomes enriched for undigested lysosomal substrates [223]. Considering the key role of VPS34 in autophagy, many compounds aim to target this kinase. The following section will present the characterization of VPS34 inhibitors Spautin-1, autophinib, SAR405, VPS34-IN1, and Cpd18.

Spautin-1 does not directly affect the catalytic activity of VPS34 but promotes the degradation of VPS34 complexes by inhibiting ubiquitin-specific peptidases USP10 and USP13. Under glucose-free conditions, Spautin-1 supports the ubiquitination of Beclin1 and triggers its degradation leading to destabilization and degradation of VPS34 complexes and inhibition of autophagy. VPS34 complexes also provide a molecular mechanism for class III PI3K to control the levels of p53. Therefore, levels of p53 are reduced in the tissues of BECN1+/− mice [224]. Exosome production was found to be regulated by the p53 response as up-regulation of TSAP6 transcription by activated p53 can increase exosome release [225]. The destabilization of the PI3K complex that occurs upon suppressing Beclin1 (either via siRNA-mediated knockdown or Spautin-1 treatment) reduced both exosome release and autophagy flux in chronic myeloid leukaemia cells [226]. Alternatively, Beclin1 may regulate autophagosome formation [227] and fusion of endosomes and autophagosomes leading to amphisome formation [228, 229]. In addition, it was also found that Beclin1 is needed for autophagosome fusion with lysosomes [230].

Autophinib is an ATP-competitive inhibitor of VPS34 decreasing the accumulation of the lipidated protein LC3 on the autophagosomal membrane. It inhibits autophagy induced by rapamycin or by amino acid starvation. The *in vitro* IC50 value for VPS34 is 19 nM [231]. Since VPS34 has also a role in the fusion of autophagosomes with lysosomes, VPS34 inhibition causes autolysosomal dysfunction, a phenotype with large late endosomes and an enhanced release of atypical exosomes harbouring poly-ubiquitinated proteins [136–138].

SAR405 and VPS34-IN1 are highly potent and selective inhibitors of VPS34 not affecting the closely related class I and class II PI3Ks. SAR405 and VPS34-IN1 cause defects in autophagosome formation and endosomal trafficking [232]. SAR405 can inhibit autophagy induced by starvation and/or mTOR inhibition [233]. SAR405 prevents the catalytic activity of ATG14L and UVRAG-containing VPS34 complexes, induces late endosome swelling, and affects late endosome-lysosome compartments [233]. Using SAR405 decreased the tumour growth and improved mouse survival in multiple tumour models by inducing tumour infiltration of NK, CD8+, and CD4+ T effector cells [96] and repressed viability of liver cancer stem cells [97]. Nevertheless, VPS34 function is critical in dendritic cells where it controls antigen cross-presenting. Consequently, VPS34 inhibition may lead to impaired T-cell–mediated immunity that may limit the use of SAR405 in anticancer therapy [99].

VPS34-IN1 selectively decreased PI3P levels, increased Beclin1 levels, but did not downregulate its other interacting partners from complex I, namely ATG14L, and VPS15 (in contrast to knockout of VPS34), ruling out indirect effects of destabilization of these proteins [223]. PI3P deficiency was shown to promote the secretion of unique exosomes enriched in undigested lysosomal substrates, including amyloid precursor protein C-terminal fragments, specific sphingolipids, and the phospholipid bis(monoacylglycero)phosphate. Secretion of these exosomes needs neutral sphingomyelinase 2 and sphingolipid synthesis. It was noted that proteins typically associated with exosomes such as Alix and CD63, or with ESCRT-dependent ILV sorting (TSG101 and Hrs) were minimally affected, suggesting that VPS34-IN1 likely affects composition rather than quantity of extracellular vesicles [223]. Neurons treated with VPS34-IN1 showed delayed degradation of EGFR following EGF stimulation. The remaining degradation was blocked by V-ATPase inhibitor bafilomycin A1, suggesting that VPS34 inhibition only partially impairs lysosomal function [223].

Cpd18 and 3-MA are structurally related compounds that differ only in a methyl piperidine group at the C6 of the adenine, but unlike 3-MA, Cpd18 does not inhibit class I PI3Ks. Cpd18 inhibits omegasome formation [234]. Nevertheless, the concentrations of Cpd18 that presented a greater attenuation of the autophagic flux are associated with cytotoxicity [210] and decreased ubiquitin/proteasome-dependent proteolysis in living cells [234].

### Late autophagy inhibitors

Another way to inhibit autophagy is to target the later stages of the autophagy machinery, such as the fusion of autophagosomes with lysosomes and the degradation of autolysosome content by the lysosomal enzymes. Autophagosome–lysosome fusion involves the action of SNAREs. Autophagosome-lysosome fusion is orchestrated by the autophagosomal SNAREs STX17 and SNAP29, lysosomal R-SNARE VAMP8, HOPS tethering complex, small GTPase RAB7, and accessory proteins such as ATG14. Interestingly, the translocation of STX17 occurs only on complete autophagosomes and not on partially formed autophagosomes. EACC blocks autophagosome-lysosome fusion by preventing STX17 and SNAP29 loading onto autophagosomes without impeding the completion of autophagosomes. It also causes an accumulation of LC3-II and reduces the interaction of STX17 with the HOPS subunit VPS33A and the lysosomal VAMP8. On the other hand, autophagy induction, the number of autophagosome biogenesis sites, expansion of the phagophore, lysosomal pH, localization of lysosomal SNAREs or RABs, and cargo recognition remain unaltered in the presence of EACC [235] although STX17 was also shown to be involved in autophagy initiation [46]. Interestingly, STX17 depletion increased the production of exosomes in A549 cells [236] and effectively blocks the formation of axonal amphisomes after 3 hours starvation in dorsal root ganglion neurons [237].

Chloroquine (CQ) and its derivatives (such as 3-hydroxychloroquine HCQ or Lys05) inhibit the maturation of autolysosomes and block late steps of autophagy. In contradiction with previous studies [238], some more recent studies indicate that CQ does not substantially decrease lysosomal acidity, and the lysosomes retain their capacity to degrade delivered material [239]. Although CQ may induce a temporal elevation of lysosomal pH, this elevation may be only transient and can be followed by reacidification of the lysosomes [240]. The kinetics of this transient phase may differ between cell types [239]. The greater part of the confusion about CQ effects on lysosomal pH might be attributed to how it was measured because LysoTracker Red dye, often used to estimate lysosomal pH, is not a pH sensor and the intensity of its fluorescence signal does not correlate with the lysosomal pH [239]. Nevertheless, CQ behaves as a weak base and accumulates in the lysosomes causing lysosomal stress. Lysosomal stress may cause the release of EVs to eliminate cellular waste [223]. Accordingly, the production of exosomes was increased due to CQ treatment [236]. CQ treatment led to marked lysosomal swelling and recruitment of Galectin-3 to sites of membrane damage [241]. In response to lysosomal damage, IL-1β can be recognized by secretory autophagy cargo receptor TRIM16 [176]. Strikingly, glucose starvation or hexokinase inhibition by 2-deoxyglucose prevented CQ from inducing lysosomal damage and subsequent cell death [241]. Accordingly, IL-1β release correlates with the degree of lysosome damage [242] and glucose is required for LPS-induced IL-1β production by monocytes [243]. Furthermore, autophagy inhibition with CQ also induced the secretion of pro-inflammatory cytokines MIF (Macrophage migration inhibitory factor) and IL-6 in triple-negative breast cancer cells [244].

Although CQ and HCQ are indisputably impairing the autophagic flux, their use entails multiple side effects including the disorganization of the Golgi and endo-lysosomal networks, dysregulation of STX17 and SNAP29 targeting, and even a temporary induction of autophagic sequestration activity (probably by inhibiting mTORC1 in a Rag-dependent manner [245]) and a drop in ATP content [227, 239]. Furthermore, during starvation or CQ-induced lysosomal stress, TFEB and TFE3 rapidly translocate to the nucleus to initiate lysosomal biogenesis [240]. CQ was also shown to increase amphisome and IFN-α production in human plasmacytoid dendritic cells stimulated by the Herpes simplex virus. On the other hand, when Beclin1 was knocked down, virus-induced IFN-α production was significantly suppressed [246].

Bafilomycin A1 (BAFA1) is a V-ATPase inhibitor that blocks the autophagic flux by inhibiting autophagosome-lysosome fusion (possibly by inhibiting the ATP2A/SERCA pump [247]) and autolysosomal and/or lysosomal acidification. It also reduces the delivery of internalized molecules from MVBs to lysosomes [248]. On the other hand, trafficking through early and late endosomes continues upon BAFA1 treatment [249]. BAFA1-treated cells display phenotypes associated with an inhibition of the degradation capacity of lysosomes such as the presence of intact cytoplasm in the lysosomal lumen and a loss of acidity [239]. BAFA1 inhibits lysosomal degradation and thereby negatively affects the amino acid efflux from the lysosomes, possibly impairing mTOR signalling which is dependent on this organelle (mTOR localizes to lysosomes and its activation depends on amino acids inside the lysosomal lumen) [245]. mTOR inhibition strongly decreased exosomal prion release [217]. In contrast, starvation stimulated exosome release through a RAB27a-dependent mechanism but did not significantly alter exosomal cargo content [250]. BAFA1 also triggers BAX- and/or BAK-dependent cytotoxicity and caspase activation [210] and has indirect effects on Golgi trafficking [251]. According to some indications, BAFA1 can also inhibit signals from the lysosomal P5-type ATPase ATP13A2 (also known as PARK9) [252]. ATP13A2 has been found to regulate both autophagic degradation and exosomal release [253]. Elevated levels of ATP13A2 enhance the externalization of α-synuclein through amphisomal structures, which is proposed to be accomplished through ATP13A2-mediated modulation of intraluminal zinc ion levels in MVBs [254, 255]. On the other hand, the exosomal release was enhanced due to BAFA1 treatment [153, 236]. Inhibition of autophagy with BAFA1 markedly reduced IL-1β secretion induced by LPS+ATP in human neutrophils [220].

The principal enzymatic activity of PIKfyve (phosphoinositide kinase, FYVE-type zinc finger containing) is to phosphorylate PI3P to PI(3,5)P2. PIKfyve inhibitors, such as YM201636, vacuolin-1, and apilimod mesylate, disrupt lysosome turnover, the heterotypic fusion of lysosomes with autophagosomes and/or MVBs, and the formation of autolysosomes resulting in autophagy inhibition [256]. Acute inhibition of PIKfyve also blocks protein sorting and their turnover in late endosomes. Both PI(3,5)P2-deficient cells and cells that lack TRPML1 exhibited enlarged endolysosomes and trafficking defects in the late endocytic pathway [257]. Furthermore, PI(3,5)P2 is required for lysosomal acidification by the V-ATPase [258]. Consequently, PIKfyve inhibition enhances exosome release and triggers secretory autophagy in PC-3 cells (probably to relieve stress caused by disruption of recycling pathways). These exosomes bear the typical exosomal markers (TSG101, Alix) and a subset of ATGs [259].

## Conclusion

Autophagy and MVBs-related secretory pathways are interconnected at many levels. These pathways, collectively with the ubiquitin-proteasome system, orchestrate the dynamics of intracellular waste removal, where each pathway may complement the deficiencies of the other. In other words, exosome secretion can reduce stress when degradative and recycling pathways are disrupted. On the other hand, unwanted MVBs with damaged material may be directed to lysosomes. Furthermore, some parts of functional autophagy machinery are important for the genesis of endosomes and amphisomes. Consequently, autophagy inhibition can both promote and/or decrease EVs release. The resulting effect is largely context-dependent and could be significantly affected by different kinds of autophagy modulators. Moreover, modulation of autophagy significantly impacts not only EVs quantity but probably also their content. Late and early autophagy inhibitors can have a profoundly different effect on secretion. For example, Spautin-1 and CQ are both autophagy inhibitors but have nearly opposite effects on EVs release. Many studies suggest that cancer cells release higher amounts of EVs compared to non-malignant cells, which makes the effect of autophagy inhibitors on EVs secretion highly important and attractive for anticancer therapy. In future studies, it should be carefully assessed how exactly autophagy could be targeted (late versus early autophagy inhibitors) to maximize patient benefit and improve cancer therapy. For safe and successful clinical use of autophagy inhibitors, we need to carefully explore the molecular mechanisms underlying the effects of autophagy on tumour progression and possibly discover all pathways affected by particular autophagy inhibitors (see Table 2).

**Table 2:** Inhibitors of autophagy

Drug	Systematic name	Known targets	Status	Limitations	References
MRT68921	N-[3-[[5-Cyclopropyl-2-[(1,2,3,4-tetrahydro-2-methyl-6-isoquinolinyl)amino]-4-pyrimidinyl]amino]propyl]-cyclobutanecarboxamide dihydrochloride	ULK1, ULK2, NUAK1	Preclinical	Mitotic dysregulation; crosstalk with endocytic pathways	[166, 202, 203, 260]
3-MA	3-Methyladenine	PI3Ks	preclinical	non-selectivity; activation of autophagy in a longer period of treatment; cytotoxicity; crosstalk with endocytic pathways	[206, 210–212]
Wortmannin	(1alpha,11alpha)-11-(Acetyloxy)-1-(methoxymethyl)-2-oxaandrosta-5,8-dieno(6,5,4-bc)furan-3,7,17-trione	PI3Ks, DNA-PK	preclinical	failed clinical translation due to drug-delivery challenges; proteosynthesis inhibition; crosstalk with endocytic pathways	[209, 211, 261]
LY294002	2-(4-Morpholinyl)-8-phenyl-4H-1-benzopyran-4-one	PI3Ks	preclinical	activation of autophagy; proteosynthesis inhibition; crosstalk with endocytic pathways	[207, 209, 211, 212]
Spautin-1	C43, 6-Fluoro-N-[(4-fluorophenyl)methyl]-4-quinazolinamine	USP10, USP13	preclinical	ROS-mediated DNA damag; Beclin1 degradation; crosstalk with endocytic pathways	[224, 228, 229, 262]
Autophinib	6-Chloro-N-(5-methyl-1H-pyrazol-3-yl)-2-(4-nitrophenoxy)-pyrimidinamine	VPS34	preclinical	pleiotropic impact of VPS34 inhibition; impaired T-cell–mediated immunity	[99, 231]
SAR405	(8S)-9-[(5-chloro-3-pyridinyl)methyl]-6,7,8,9-tetrahydro-2-[(3R)-3-methyl-4-morpholinyl]-8-(trifluoromethyl)-4H-pyrimido[1,2-a]pyrimidin-4-one	VPS34	preclinical	pleiotropic impact of VPS34 inhibition; defects in endosomal trafficking; impaired T-cell–mediated immunity	[99, 232, 233]
VPS34-IN1	1-((2-((2-chloropyridin-4-yl)amino)-4'-(cyclopropylmethyl)-[4,5'-bipyrimidin]-2'-yl)amino)-2-methylpropan-2-ol	VPS34	preclinical	pleiotropic impact of VPS34 inhibition; defects in endosomal trafficking; impaired T-cell–mediated immunity	[99, 232]
Cpd18	3-methyl-6-(3-methylpiperidin-1-yl)-3H-purine	omegasomes	preclinical	toxicity; decreased ubiquitin/proteasome-dependent proteolysis	[210, 234]
Chloroquine	4-N-(7-chloroquinolin-4-yl)-1-N,1-N-diethylpentane-1,4-diamine	autolysosomes; lysosomes; endosomes	21 clinical trials phase 1 or 2; only one phase 3 study (NCT00224978)	lysosomal stress; crosstalk with endocytic pathways; uptake may differ based on pH	[223, 236, 241, 244, 246, 263]
HCQ	2-[4-[(7-chloroquinolin-4-yl)amino]pentyl-ethylamino]ethanol	autolysosomes; lysosomes; endosomes	94 clinical trials; only one phase 2/3 study (NCT03008148)	uptake may differ based on pH	[227, 239, 264]
Bafilomycin A1	(3Z,5E,7R,8S,9S,11E,13E,15S,16R)-16-[(2S,3R,4S)-4-[(2R,4R,5S,6R)-2,4-dihydroxy-5-methyl-6-propan-2-yloxan-2-yl]-3-hydroxypentan-2-yl]-8-hydroxy-3,15-dimethoxy-5,7,9,11-tetramethyl-1-oxacyclohexadeca-3,5,11,13-tetraen-2-one	V-ATPase	preclinical	cytotoxicity and caspase activation; effects on Golgi trafficking; crosstalk with endocytic pathways	[210, 251, 265]
YM201636	6-amino-N-[3-(6-morpholin-4-yl-8-oxa-3,5,10-triazatricyclo[7.4.0.02,7]trideca-1(9),2(7),3,5,10,12-hexaen-4-yl)phenyl]pyridine-3-carboxamide	PIKfyve	preclinical	block of protein sorting; crosstalk with endocytic pathways and exocytosis	[257, 259]
Vacuolin-1	2-N-[(E)-(3-iodophenyl)methylideneamino]-6-morpholin-4-yl-4-N,4-N-diphenyl-1,3,5-triazine-2,4-diamine	PIKfyve	preclinical	block of protein sorting; crosstalk with endocytic pathways and exocytosis	[257, 259]
Apilimod mesylate	methanesulfonic acid;N-[(E)-(3-methylphenyl)methylideneamino]-6-morpholin-4-yl-2-(2-pyridin-2-ylethoxy)pyrimidin-4-amine	PIKfyve	preclinical	block of protein sorting; crosstalk with endocytic pathways and exocytosis	[257, 259]
EACC	ethyl (2-(5-nitrothiophene-2-carboxamido) thiophene-3-carbonyl) carbamate.	STX17	preclinical	crosstalk with endocytic pathways and exocytosis	[236, 237]

## Data Availability

Not applicable

## Abbreviations

3-MA: 3-methyladenine
AKT: Rac-alpha serine/threonine-protein kinase
Alix: Apoptosis-linked gene 2-interacting protein X
AMBRA1: Autophagy and Beclin 1 regulator 1
AMPK: AMP-activated protein kinase
ANXA2: Annexin 2
Arf6: ADP ribosylation factor 6
ATG: Autophagy-related genes
ATP: Adenosine triphosphate
ATP13A2: ATPase cation transporting 13A2
Beclin1: Coiled-coil, moesin-like BCL2 interacting protein
Bif-1: Bax-interacting factor 1
BMDC: Bone marrow-derived dendritic cells
CaMKKβ: Calcium/calmodulin-dependent protein kinase kinase β
CCL: C-C motif chemokine ligand
COPII: Coat protein complex II
CQ: Chloroquine
CSF3/G-CSF: Granulocyte colony-stimulating factor
c-Src: Proto-Oncogene Tyrosine-Protein Kinase Src
CXCL: C-X-C motif chemokine ligand,
DEPTOR: DEP domain-containing mTOR interacting protein
DFCP1: Double FYVE-containing protein 1
EGFR: Epidermal growth factor receptor
EMT: Epithelial to mesenchymal transition
ER: Endoplasmic reticulum
ESCRT: Endosomal sorting complexes required for transport
EVs: Extracellular vesicles
FIP200: FAK family-interacting protein of 200 kDa
G3BP: Ras GTPase-activating protein SH3-domain-binding protein
GDP: Guanosine diphosphate
GTP: Guanosine triphosphate
GTPase: Guanosine triphosphatase
HCQ: Hydroxychloroquine
HOPS: Homotypic fusion, and protein sorting complex
CHMP: Charged multivesicular body protein
iBMM: Bone marrow-derived macrophages
IFN-α: Interferon alpha
IKKβ: IκB kinase
IL: Interleukin
ILVs: Intraluminal vesicles
ISG15: Interferon (IFN)-α/β-induced ubiquitin-like protein
LAMP2: Lysosome-associated membrane protein 2
LC3: Microtubule-associated protein 1A/1B-light chain 3
LKB1: Liver kinase B1
LPS: Bacterial lipopolysaccharides
MGRN1: Mahogunin ring finger 1
MHC: Major histocompatibility complex (MHC-II)
MIF: Macrophage migration inhibitory factor
mLST8: Mammalian lethal with Sec13 protein 8, alias GβL
MMP: Matrix metalloproteinase
mTOR: Mammalian target of rapamycin
mTORC: Mammalian target of rapamycin complex
MVBs: Multivesicular bodies
NF-κB: Nuclear factor-kappa B
NUAK1: NUAK family SNF1-like kinase 1
p115: Vesicular transport factor p115
PARK7: Parkinsonism-associated deglycase 7
PI(3,4,5)P3: Phosphatidylinositol (3,4,5)-trisphosphate
PI3K: Phosphoinositide 3-kinase
PI3P: Phosphatidylinositide-3-phosphate
PIKfyve: Phosphoinositide kinase, FYVE-type zinc finger containing
PKA: Protein kinase A
PLD2: Phospholipase D2
PRAS40: Proline-rich Akt substrate of 40 kDa
Rab: Ras-associated GTP-binding protein
Rag: Ras-related GTP-binding protein
RAPTOR: Regulatory-associated protein of mTOR also known as KIAA1303
Rheb: Ras homolog enriched in brain
SEC22b: SEC22 vesicle-trafficking protein homolog B
shRNA: Small hairpin RNA
siRNA: Small interfering RNA
SNAP29: Synaptosome-associated protein 29
SNARE: Soluble N-ethylmaleimide-sensitive fusion attachment protein receptors
STAT3: Signal transducer and activator of transcription 3
STX17: Syntaxin-17
TBC-2: TBC domain-containing protein 2
TFE3: Transcription factor binding to IGHM Enhancer 3
TFEB: Transcription factor EB
TME: Tumour microenvironment
TREM1: Triggering receptor expressed on myeloid cells 1
TRPML1: Transient receptor potential channel mucolipin 1
TSC: Tuberous sclerosis complex
TSG101: Tumuor susceptibility 101
ULK: Unc51-like autophagy-activating kinase
UVB: Ultraviolet light B
UVRAG: UV resistance-associated gene
VAMP: Vesicle-associated membrane protein
V-ATPase: V1V0-vacuolar-type ATPase
VPS: Vacuolar protein sorting
WIPI: WD repeat domain phosphoinositide-interacting protein
γ-H2AX: Phosphorylation of the Ser-139 of the histone variant H2AX
